# The Orthopædics of the Future: A New Book by Professor Spitzy
**The Diseases of Children.* Edited by Prof. M. Pfaundler and Prof. A. Schlossmann. Vol. V. Congenital Diseases: Surgery, Orthopædics. By Dr. Hans Spitzy. Translated by H. Shaw, M.D., and. L. La Fetra. J. B. Lippincott Co., Philadelphia and London. Price 21s. net. Pp.364. 865 illustrations.


**Published:** 1912-07-13

**Authors:** 


### THE ORTHOPÆDICS OF THE FUTURE.

### A New Book by Professor Spitzy.*

Graz is a picturesque, pine-environed town, the capital of Styria, one of the less well-known counties of Austria. A city of some 200,000 inhabitants, it boasts much that is of general interest to the tourist. The artist will find in it delightful alley-ways, quaint cornices, entablatures, and colonnades, over which the fantastic influence of Oriental architecture has been thrown in a manner that still appeals. The lover of natural scenery will come across unrivalled bits of forest, wood, and mountain land in the neighbourhood of the town, through which the murmuring river Mur, vocal a mile beyond sight, foams with the impetuousness of a hillside spate. And last, but not least, the post-graduate student, in search of instruction, novelty, interest in matters that appeal to him, will at Graz light upon fields that are singularly rich in professional attractions.

There is the fine new surgical clinic, perhaps the richest one-man-controlled hospital in the world, where Professor von Hacker, the youngest of Bilroth's pupils, and well known as the originator of posterior gastro-enterostomy, still lectures and operates, most ably assisted by Professor Streissler, who has won fame as the exponent of bone transplantation. There is the large medical department of the General Hospital, the school which has given to Munich, Erlangen, and Berlin some of its finest teachers. There is the up-to-date hospital of the Brothers of Charity, the surgical side of which is a model of voluntary hospital control. And above all there is the Anna Kinderspital where, up to very recently, the surgical department was in charge of one of the best known orthopædists of the day, Professor Hans Spitzy. To orthopædists, Spitzy's name is well known, and his sound work in nerve surgery, of which he is one of the pioneers, has given him a world-wide reputation.

### The Man and His Work.

A native of Graz, Dr. Spitzy has preferred to work here, and at this comparatively small children's hospital to carry out experimental and clinical work, which in the department in which he specialises may almost be said to be epoch-making. His hospital is one of the recognised places which the orthopædist is expected to visit. No one who has any claim to being considered a good orthopædist has neglected to go there to see Spitzy and study his methods. The favourite pupil of the well-known Hoffa, and a disciple of the no less famous Nicoladoni, probably one of the most brilliant operative surgeons who has ever lived, Spitzy accompanied Hoffa to Berlin as first assistant, and later on to America, where he was promptly offered a professorship of surgery at an American University. But he preferred to remain with his chief, and at the latter's death established himself at Graz, where he soon won a recognised position by his writings and work. To post-graduates he has always been a cordial friend. Speaking English remarkably well, and well acquainted with French and Italian, his clinic was a favourite meeting place for a cosmopolitan gathering of practitioners attracted thither by his reputation. Always courteous, hospitable, and kind, he made them feel at home, and many when they left Graz did so with a sense of real regret that their departure broke, for the time being at least, the bond of personal friendship so quickly formed by this virile, confident, black-moustached young man.

Now Dr. Spitzy has left Graz to take up work at the General Hospital at Vienna, which offers a large scope for his genius. Before he went he published a book which may be taken as a farewell monograph compiled from material at the Anna Children's Hospital. As such it has a permanent interest to everyone who worked under him there. To some of us, who are familiar with the cases recorded in these pages, the work will appeal more powerfully than to others who accept the book merely as a clinical text-book. But to all it will appeal as probably one of the finest works on orthopædics which has been published in the English language. The translation, with one or two small slips excepted, has been very well done, and publishers and printers alike are to be congratulated on the excellent get-up of the work.

### The Newest Work on Orthopædics.

A few chapters are contributed by Professor Lange, but the bulk of the work belongs to Spitzy. And it is singularly good work. We are not given an epitome of text-book opinions, a rewritten record of what has been said, often on the flimsiest evidence, by other men. We are given, on the contrary, the actual results of personal clinical experience, told in a succinct, clear, and often very attractive manner. The writer's style is here and there too abrupt, almost staccato, but it is due to the fact that he restrains himself and never commits himself to an opinion unless he has excellent grounds for it. It is this cautiousness, conjoined with the mass of really valuable practical hints—just those hints which the student cannot glean from ordinary text-books—that will make this book so useful and informative to practitioners who desire to know something about the advancement of modern orthopædics.

No chapter in modern surgery reads so magnificently as this—the development of modern treatment of deformities. A few years ago, well within the memory of most of us, an open operation, with a high mortality attaching to it, was the only hope for the child suffering from congenital dislocation of the hip—a hope which was often doomed to disappointment, since the results of operation were most unsatisfactory. Now the cure of this deformity

* *The Diseases of Children.* Edited by Prof. M. Pfaundler and Prof. A. Schlossmann. Vol. V. Congenital Diseases: Surgery, Orthopædics. By Dr. Hans Spitzy. Translated by H. Shaw, M.D., and L. La Fetra. J. B. Lippincott Co., Philadelphia and London. Price 21s. net. Pp. 364. 865 illustrations.

can be undertaken by any general practitioner who has studied the methods of manual reposition of Paci, Schede, and Lorenz. Formerly the boy who limped owing to the deformity occasioned by coxa vara was doomed to go through life wearing apparatus or trudging dot-and-carry-one without. Now, thanks to the efforts of Hoffa, Lovett, and Lange, the deformity can be cured with comparatively little danger to life or limb. Even nowadays many common deformities—we may instance flat feet and bowed legs—are treated on antiquated lines which the exponents of the new orthopædy have shown to be absolutely fallacious. The profession learns slowly: in England especially there is a tendency to disregard foreign advances, just as there is a slowly dying tendency to consider that every surgeon is competent to deal with a deformity and to deprecate as needless elaboration the desire for specialisation in orthopædics. Yet, reading this book and realising what has been done, who can justify that tendency? In the case of nervous lesions, paralytic deformities, Dr. Spitzy has himself been the pioneer in this line, and his methods are worth careful study, for he has availed himself of the best aids, with the result that his technique is now far superior to anything in England. We may mention his rejection of the Carlile tissue for covering nerve junctions in favour of the arterial sheath as recommended by Foramitti. It is to this careful attention to asepsis, technique, and after-treatment that he owes the striking successes which he has obtained and of which the pictures in this book give such elucidative illustrations. We may specially mention the "before" and "after" photographs on pages 331, 332, and 324, showing the results of nerve anastomosis in upper arm paralysis cases. Dr. Spitzy has not given an account of his useful and highly original osteoplastic operation in cases of intractable Erb's paralysis, the principle of which consists in rearranging the abductors of the shoulder by altering the fulcrum of certain muscle levers. The operation was described in 1907 by a post-graduate student in the following words:—

Perhaps the most interesting operation, apart from the purely nervous work, one sees at Spitzy's clinic is his osteotomy of the upper end of the humerus for the correction of certain shoulder and upper arm defects. The best illustration of this novel method will be a brief description of the two cases which were done lately, and which I was able to observe carefully as to the immediate result. The first was the case of an intelligent little girl with incomplete Erb's paralysis. The supra-scapular nerve was affected, the paralysed group being the outward rotators, the spinati and the teres minor. The result was a complete inability actively to rotate the shoulder outwards, to touch the side of the face with the finger tips, or to perform any of the movements which are requisite for such a combination as obtains in "military saluting." The involvement of the teres minor pointed to some degeneration in the circumflex, but had very little to do with this loss of abducting power, since the main abductors are the spinati. The first thing here was to attempt a nerve plasty. At the operation, however, this was found to be impossible, the supra-scapular being involved in a mass of fibrous tissue and itself reduced to a mere fibrous remnant. Central implantation offered no hope of success here, and the supra-spinous wound was accordingly closed, and an incision was made on the outer side of the upper arm. The humerus shaft was exposed and chiselled through below the deltoid insertion. The lower fragment was then strongly rotated outwards, and accurately approximated to the upper fragment in this position. The arm was put up abducted to a right angle with the shoulder, in strong outward rotation, with flexion of the elbow-joint, and immobilised in an accurately fitting plaster bandage. Passive movement was begun early, for the patient made a rapid recovery, the wounds healing by primary union. At present the child's condition is eminently satisfactory. The arm hangs comfortably by the side, and there is a strong mass of callus to be felt at the line of section of the bone. The palm of the hand looks forwards and outwards, owing to the strong external rotation which the shaft of the humerus has undergone, and in which it has firmly united, but except for this it would be difficult for an observer unacquainted with the case to say what has been the matter with the child. To all intents and purposes abduction and external rotation at the shoulder-joint are normal, but this is, of course, by no means the case, as the muscles concerned in these movements are still paralysed. What has been done is that the arm has been passively rotated outwards, and the deltoid now acts, together with the biceps, much more strongly as an abductor.

Interesting, too, are the operations for switching the "nerve current" from one muscle group to another, an expedient which was first suggested by Hoffa as an alternative to his own pronator-supinator exchange, but first actually tried, we believe, by Spitzy. Specially worthy of attention, too, is Professor Spitzy's account of his treatment in such common children's lesions as prolapsus ani (which he cures by means of his wax injections, the technique of which is lucidly described, with photographs showing the actual procedure) and empyema. The chapter on appendicitis in children—for the book is not entirely devoted to orthopædics, but to general pedriatic surgery—is one of the clearest and best accounts of the surgical treatment we have met with. Equally good is the article dealing with injuries. But the main interest of the book centres round the deformities, especially those of nervous origin. The future of orthopædics is the future of nerve surgery—using the term surgery not so much to imply operation (for the good orthopædist only operates as a last resource) as manipulation. This is well illustrated in the altered conception of the essentials of treatment in cases of anterior poliomyelitis (to use an old but bad name). Professor Spitzy's book is undoubtedly one of the best works on the surgery of children's diseases we possess: on the orthopædic aspect of the matter it is unsurpassed for practical utility and sound teaching.

